# Sex-Based Differences in Cardiac Gene Expression and Function in BDNF Val66Met Mice

**DOI:** 10.3390/ijms22137002

**Published:** 2021-06-29

**Authors:** Marcus Negron, Jeffrey Kristensen, Van Thuan Nguyen, Lauren E. Gansereit, Frank J. Raucci, Julia L. Chariker, Aaron Heck, Imamulhaq Brula, Gabrielle Kitchen, Cassandra P. Awgulewitsch, Lin Zhong, Eric C. Rouchka, Simran Banga, Cristi L. Galindo

**Affiliations:** 1 Department of Biology, Western Kentucky University, Bowling Green, KY 42101, USA; marcus.negron447@topper.wku.edu (M.N.); jeffrey.kristensen381@topper.wku.edu (J.K.); vanthuan.nguyen@wku.edu (V.T.N.); lauegans@ut.utm.edu (L.E.G.); aaron.heck858@topper.wku.edu (A.H.); imamulhaq.brula212@topper.wku.edu (I.B.); gabrielle.kitchen429@topper.wku.edu (G.K.); simran.banga@wku.edu (S.B.); 2 Department of Pediatrics, Division of Pediatric Cardiology, Children’s Hospital of Richmond, Virginia Commonwealth University, Richmond, VA 23219, USA; rauccifj@mymail.vcu.edu (F.J.R.); lin.zhong@vumc.org (L.Z.); 3 Computer Engineering and Computer Science, Kentucky Biomedical Research Infrastructure Network, University of Louisville, Louisville, KY 40202, USA; julia.chariker@louisville.edu (J.L.C.); Eric.rouchka@louisville.edu (E.C.R.); 4 Vanderbilt University Medical Center, Division of Cardiovascular Medicine, Nashville, TN 37232, USA; cassandra.p.awgulewitsch@vanderbilt.edu

**Keywords:** brain-derived neurotrophic growth factor, dilated cardiomyopathy, rs6265 polymorphism, Duchenne muscular dystrophy, Val66Met

## Abstract

Brain-derived neurotrophic factor (BDNF) is a pleiotropic neuronal growth and survival factor that is indispensable in the brain, as well as in multiple other tissues and organs, including the cardiovascular system. In approximately 30% of the general population, BDNF harbors a nonsynonymous single nucleotide polymorphism that may be associated with cardiometabolic disorders, coronary artery disease, and Duchenne muscular dystrophy cardiomyopathy. We recently showed that transgenic mice with the human BDNF rs6265 polymorphism (Val66Met) exhibit altered cardiac function, and that cardiomyocytes isolated from these mice are also less contractile. To identify the underlying mechanisms involved, we compared cardiac function by echocardiography and performed deep sequencing of RNA extracted from whole hearts of all three genotypes (Val/Val, Val/Met, and Met/Met) of both male and female Val66Met mice. We found female-specific cardiac alterations in both heterozygous and homozygous carriers, including increased systolic (26.8%, *p* = 0.047) and diastolic diameters (14.9%, *p* = 0.022), increased systolic (57.9%, *p* = 0.039) and diastolic volumes (32.7%, *p* = 0.026), and increased stroke volume (25.9%, *p* = 0.033), with preserved ejection fraction and fractional shortening. Both males and females exhibited lower heart rates, but this change was more pronounced in female mice than in males. Consistent with phenotypic observations, the gene encoding SERCA2 (*Atp2a2*) was reduced in homozygous Met/Met mice but more profoundly in females compared to males. Enriched functions in females with the Met allele included cardiac hypertrophy in response to stress, with down-regulation of the gene encoding titin (*Tcap*) and upregulation of BNP (*Nppb*), in line with altered cardiac functional parameters. Homozygous male mice on the other hand exhibited an inflammatory profile characterized by interferon-γ (IFN-γ)-mediated Th1 immune responses. These results provide evidence for sex-based differences in how the BDNF polymorphism modifies cardiac physiology, including female-specific alterations of cardiac-specific transcripts and male-specific activation of inflammatory targets.

## 1. Introduction

Brain-derived neurotrophic factor (BDNF) is the most abundant member of the nerve growth factor (NGF) family and critical for neuronal growth and function. As with other NGF members, BDNF is produced as a pro-protein that can be cleaved before or after secretion by proconvertases [1,2], furin [3] plasmin [4], or matrix metalloproteinases [5], releasing roughly similarly sized mature BDNF (mBDNF) and prodomain regions [6,7]. BDNF can evoke either active or passive signaling, which is complex, as well as context- and cell type-dependent. ProBDNF binds with low affinity to a common NGF receptor (p75^NTR^) [8,9], which dimerizes with sortilin and generally leads to apoptosis [10], a process important for neuronal pruning, especially during development [7,11,12,13]. Upon cleavage, mBDNF binds its specific tyrosine kinase receptor (TrkB) with high affinity, which homodimerizes and is phosphorylated, leading to the induction of three canonical signaling pathways (PI3K/ERK/PLCy) with generally “positive” outcomes for neuronal and other cell types [14,15,16]. The prodomain serves as a chaperone for mBDNF to facilitate trafficking and secretion [17] but may also serve as an active ligand for the sortilin-related Vps10p-domain sorting receptor 2 (SorCS2) [18]. Isolated murine cardiomyocytes were recently shown to produce and respond to BDNF via a truncated TrkB isoform [19,20], which unlike the full-length isoform lacks the kinase domain and was thus originally thought to function only as a negative “sink” for mBDNF signaling [21,22]. However, cardiomyocyte-specific deletion of the truncated isoform of TrkB impairs calcium signaling, cardiac contraction, and regulation of Ca(^2+^)/calmodulin-dependent protein kinase II (CAMKII) activity [20]. The mechanisms involved are poorly understood, but increasingly there is evidence to support a critical role for BDNF in the cardiovascular system and associated diseases [23].

BDNF and its receptor are essential for heart development, microvasculature development, and atrial septation [24]. BDNF also regulates heart rate via brainstem cholinergic parasympathetic neurons [25] and is a critical neurotrophin for cardiac efferent nerves, as demonstrated by altered cardiac remodeling in response to systemic genetic disruption of BDNF or TrkB in a mouse myocardial injury model [26]. Nonetheless, the mechanisms involved in BDNF-mediated regulation of cardiovascular functions are poorly understood. In humans, circulating BDNF has been correlated with a variety of pathological conditions, including some cardiovascular disorders [27,28,29,30,31]. These findings are complicated by the fact that the primary source of circulating BDNF is platelets [32]. Altered BDNF levels in serum relating to cardiac distress are thus possibly attributable to an indirect and generalized systemic response and not organ dysfunction per se.

In ~30–70% of the general population, depending on region, BDNF harbors a nonsynonymous nucleotide polymorphism (rs6265) in which a guanine is altered to an adenine, consequently resulting in an amino acid change from a valine to a methionine at codon 66 in the prodomain of the BDNF protein [33]. This change alters BDNF trafficking and secretion, lowering the effective bioavailability of mBDNF at the synaptic cleft, neuromuscular junction, and similar paracrine signaling regions [33,34]. This Val66→Met polymorphism may also alter proBDNF signaling and confer unique prodomain-mediated signaling [17,35], although the mechanisms involved are not well-characterized. Few studies have examined the effects of the rs6265 BDNF polymorphism on the heart [36,37], but we previously found that cardiomyocytes isolated from transgenic Val66Met mice exhibit reduced contractility [38]. These mice were bred in heterozygous pairs for these prior studies, which focused on recessive, X-linked Duchenne muscular dystrophy (DMD) and thus were limited to male mice. We nonetheless evaluated females as a matter of experimental course, because the scientific community recognizes the historical exclusion of female mice to be detrimental to equality of biomedical advancements for women [39]. We hypothesized that the BDNF rs6265 polymorphism exerts subtle molecular signaling modifications that exacerbate overt cardiac comorbidities independent of sex. However, based on anecdotal observations, we serendipitously noted differences in males and females sufficient to warrant further investigation. In this study, we sought to determine the global gene expression patterns that might account for differential cardiac function, as well as to compare male and female Val66Met mice directly. Here we present evidence that the rs6265 Val66Met mutation alters the baseline cardiac transcriptome and is sexually dimorphic in the context of normal physiological conditions.

## 2. Results

### 2.1. Left Ventricular Function in BDNF Val66Met Mice

**Figure 1:**
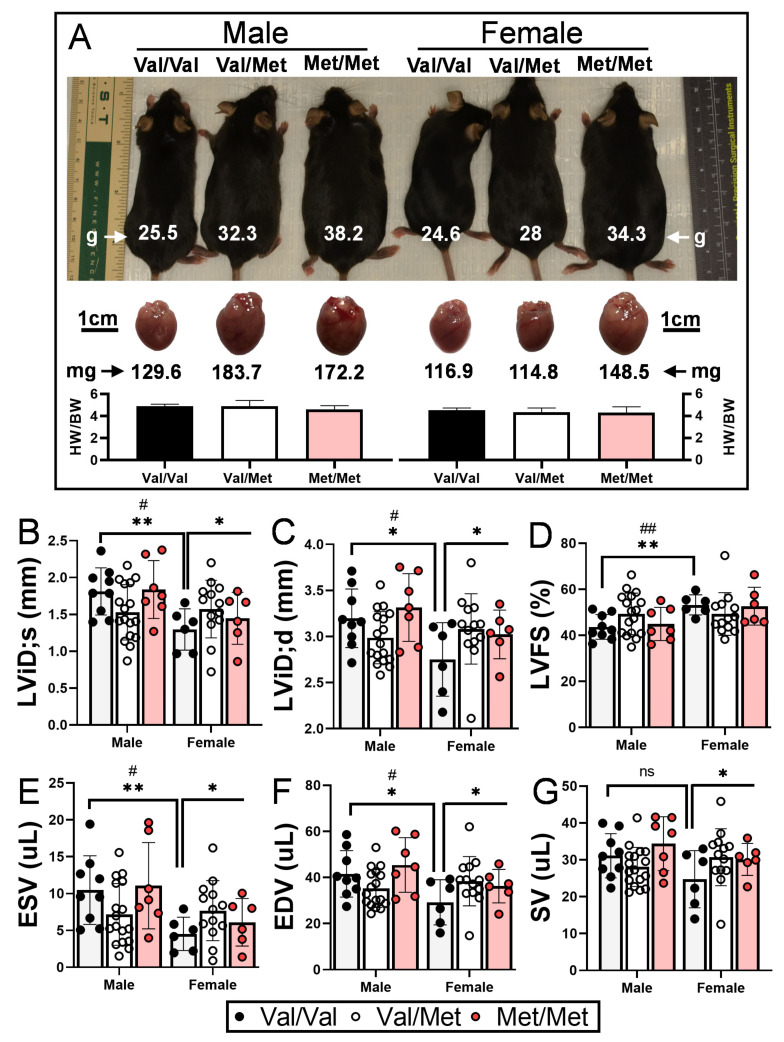
Val66Met leads to increased end systolic and diastolic volumes in female mice. (**A**) Representative 12-week-old littermate mice along with excised hearts from the same mice, showing greater body and heart weights in both males and females with the Met allele. Bars beneath representative hearts show similar heart weight-to-body weight ratios (HW/BW) among genotypes (*n* = 4 per group). (**B**–**G**) Plot showing results of echocardiography assessment of littermate-matched male and female mice (8–12 weeks) with one (Val/Met, white bars and circles) or both (Met/Met, red bars and circles) of the rs6265 alleles, relative to non-carrier controls (light gray bars and black circles). Parameters shown (*y* axis) are (**B**) left ventricular internal dimension at end systole (LVID;s) and (**C**) diastole (LVID;d), (**D**) percent (%), fractional shortening (FS), (**E**) end systolic volume (ESV), (**F**) end diastolic volume (EDV), and (**G**) stroke volume (SR). *n* = 9 (Male Val/Val), *n* = 17 (Male Val/Met), *n* = 7 (Male Met/Met), *n* = 6 (Female Val/Val), *n* = 13 (Female Val/Met), *n* = 6 (Female Met/Met). Asterisks represent statistical significance between male and female Val/Val mice or between Val/Val versus Val/Met and Met/Met female mice. * *p* < 0.05and ** *p* < 0.001 using two-way ANOVA. ^#^ *p* < 0.05 and ^##^ *p* < 0.01 using Mann–Whitney, ns = not significant.

As has been previously demonstrated, both male and female Met/Met mice weigh considerably more than littermate controls [40], and accompanying heart weights are accordingly larger (Figure 1A). Wild-type (Val/Val) female mice had significantly smaller left ventricular end systolic and diastolic internal dimensions and volumes than their male counterparts (Table 1), consistent with their smaller size (Figure 1A) and what has been previously reported [41]. Although not statistically significant (*p* = 0.122), female Val/Val stroke volume (25 μL ± 8 μL) was also smaller than that of male Val/Val mice (31 μL ± 6 μL), whereas heart rates of male and female mice were essentially the same (681 and 689 bpm, respectively). These same echocardiographic parameters, however, were increased in female littermates with one or both Met alleles, resulting in left ventricular functional measures essentially on par with their male counterparts (Table 1). In contrast to male mice, for example, female mice with one or both Met alleles had significantly larger left ventricular end diastolic internal dimension (+10.7%, from 2.75 ± 0.40 mm to 3.08 ± 0.29 mm, *p* = 0.037, Figure 1B), end systolic (+41%, from 4.5 ± 0.23 to 7.7 ± 1.9 μL, *p* = 0.049, Figure 1E) and diastolic (+23.8%, from 29.9 ± 9.8 to 38.4 ± 7.5 μL, *p* = 0.037, Figure 1F) volumes, and stroke volume (+19.5%, from 24.7 ± 7.8 to 30.7 ± 6.0 μL, *p* = 0.044, Figure 1G). Although not statistically significant, left ventricular end systolic dimension was also larger for Met/Met mice compared to Val/Val littermate controls (+17.7%, from 1.30 ± 0.28 to 1.57 ± 0.23 μL, *p* = 0.69). Heart rate was a notable exception to sex-dependent differences between Met carriers and wild-type mice, as it was reduced for both males and females (Table 1). Ejection fraction and fractional shortening were similar among all three genotypes of both males and females (Table 1 and Figure 1).

### 2.2. Global Cardiac Gene Expression in BDNF Val66Met Mice

**Figure 2:**
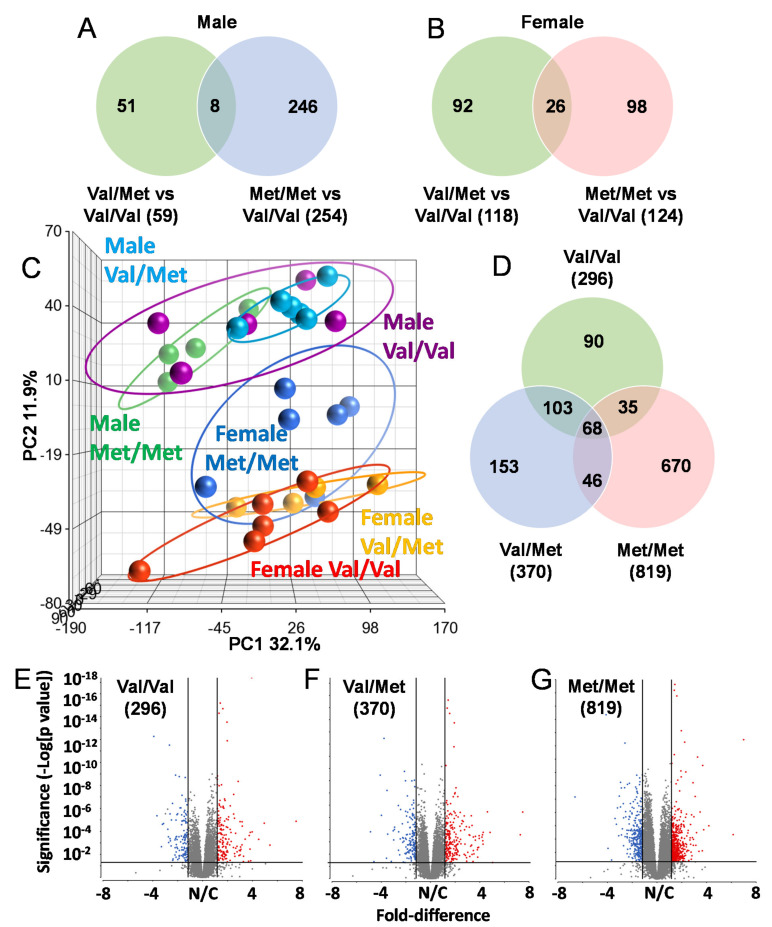
The Met allele increases sex-based differences in global cardiac transcriptional profiles. Venn diagrams showing overlap of differentially expressed genes in whole hearts of male (**A**) and female (**B**) Val/Met versus Val/Val mice compared to those altered in Met/Met versus Val/Val mice. (**C**) Principal components analysis (PCA) shows unique whole heart male and female expression profiles in Val66Met mice. Groups are as indicated and colored as follows: purple = Male Val/Val, teal = Male Val/Met, green = Male Met/Met, red = Female Val/VaWe did not use any hyphensl, orange = Female Val/Met, blue = Female Met/Met. (**D**) Venn diagram showing overlap in differential genes for direct comparisons of males versus females for each genotype (green = Val/Val, blue = Val/Met, red = Met/Met). (**E**–**G**), Volcano plots for males versus females for each genotype. The ordinate represents increasing significance, and the abscissa shows magnitude of change. Lines represent significance cutoffs, with numbers of genes significantly differentially expressed shown in parentheses. Red and blue dots represent significant genes more highly expressed in whole hearts of females and males, respectively. Grey dots represent genes not deemed as significantly different (*p* > 0.05 and/or fold < 1.5).

To identify possible mechanisms that could account for altered cardiac functions in mice with the human BDNF rs6265 polymorphism, we performed RNA-Seq analysis of whole hearts from all three possible genotypes of both male and female Val66Met mice. All mice were age-matched (8 weeks old) and litter-matched in batches. Strikingly, there was very little overlap in differentially expressed genes in Val/Met and Met/Met mice when compared to normal controls, irrespective of sex (Figure 2). In males, there were 59 and 254 differentially expressed genes, only 8 of which were commonly altered (Figure 2A). These included unclassified non-coding RNA genes, pseudogenes and only one characterized transcript (complement factor D), which was significantly down-regulated in both Val/Met (*p* = 1.4 × 10^−4^, 4.7-fold) and Met/Met FDR (*p* = 5.6 × 10^−7^, 5-fold) mice compared to Val/Val littermate controls. Similarly, genes with differential expression in female Val/Met and Met/Met mice, compared to littermate (Val/Val), were mostly non-overlapping (Figure 2B). Most of these genes were also uncharacterized with notable exceptions including up-regulation of calcium binding protein 1 (*Cabp1*, 3.1 × 10^−9^–2.2 × 10^−5^, 1.5–1.8-fold) and down-regulation of adenosine A2b receptor (*Adora2b*, 5.1 × 10^−5^–2.3 × 10^−3^, 1.5–1.8-fold).

Initial observations of global profiles showed that male and female samples were distinguishable, based on principal components analysis (PCA, Figure 2C). However, whereas heart samples of male Val/Met (Figure 2C, teal circles) and Met/Met mice (Figure 2C, green circles) were distinguishable as sub-clusters within the larger cluster including Val/Val (Figure 2C, purple circles), female Met/Met (Figure 2C, blue circles) samples clustered apart from Val/Val and Val/Met littermate-matched samples (Figure 2C, red and yellow circles, respectively). Consistent with known differences in cardiac expression between males and females, there were 296 genes differentially expressed between wild-type (Val/Val) male and female mice Figure 1D,E). The addition of the rs6265 allele remarkably increased these differences to 370 genes for female versus male Val/Met heterozygous mice (Figure 1F) and 890 genes for homozygous mutants (Figure 1G). As with intra-sex comparisons, most genes were unique for each of the groups considered (i.e., 90 unique to normal mice, 153 unique to Val/Met mice, and 670 differentially expressed only in Met/Met mice, Figure 1E).

### 2.3. Functional Analysis of Homozygous Mutants

**Figure 3:**
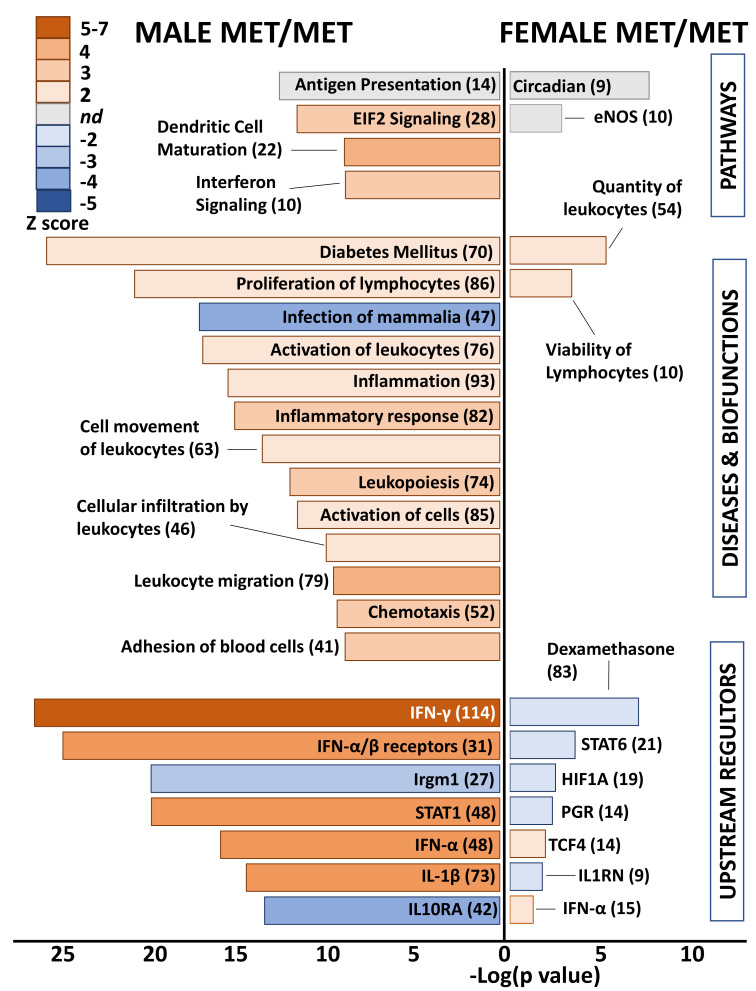
Met/Met mice exhibit a male-specific inflammatory profile. Bar chart shows enriched pathways (**top** bars), diseases and biofunctions (**middle** bars), and predicted upstream regulators (**bottom**) for whole hearts of male (**left** side) and female (**right** side) Met/Met mice compared to those of normal Val/Val controls. Bar size reflects significance (shown on *x* axis), and color represents Z score (significance of prediction of directionality), as indicated in the legend. Numbers in parentheses indicate numbers of genes in the listed category that were altered in hearts of either male or female Met/Met mice compared to respective controls.

Differential genes in whole hearts of homozygous (Met/Met) male mice were overwhelmingly related to immune responses and inflammation (Figure 3). Enriched pathways included antigen presentation, EIF2 signaling, dendritic cell maturation, and integrin signaling. The top disease category was diabetes mellitus, which was predicted to be activated. All remaining diseases and biofunctions were immune related, including proliferation of lymphocytes, inflammatory response, leukocyte activation and migration, and chemotaxis. Consistent with inflammatory activation, the category “infection of mammalia” was predicted to be inhibited based on gene expression changes in whole hearts of males with the Met allele, as compared to littermate Val/Val control mice. Upstream regulators that were predicted as activated in male Met/Met mice compared to controls were interferons (IFN-γ, IFN-α and its receptors), interleukin-1β (IL-1β), and signal transducer and activator of transcription 1 (STAT1). Conversely, immunity related GTPase M (Irgm1) and IL-10 receptor α (IL10RA) were inhibited in male Met/Met hearts versus hearts of wild-type mice.

In contrast to the immune related functional classification of genes differentially expressed in male Met/Met hearts versus those of Val/Val mice, no obvious functional pattern was detected in whole hearts of female mice with the Met allele. Top canonical pathways were circadian rhythm and eNOS signaling pathway, although directionality (activation or inhibition) was not determined. Number and viability of leukocytes were predicted as activated biofunctions, and as well as the upstream regulator IFN-α, but this functional pattern of immune-response gene expression was muted in comparison to male littermates. STAT6, the master transcription regulator of hypoxic stress (hypoxia inducible factor 1 α, HIF1A), the receptor for progesterone (PGR), IL1 receptor antagonist (IL1RN), and dexamethasone were predicted to be inhibited.

### 2.4. Functional Analysis of Heterozygous Mice

**Figure 4:**
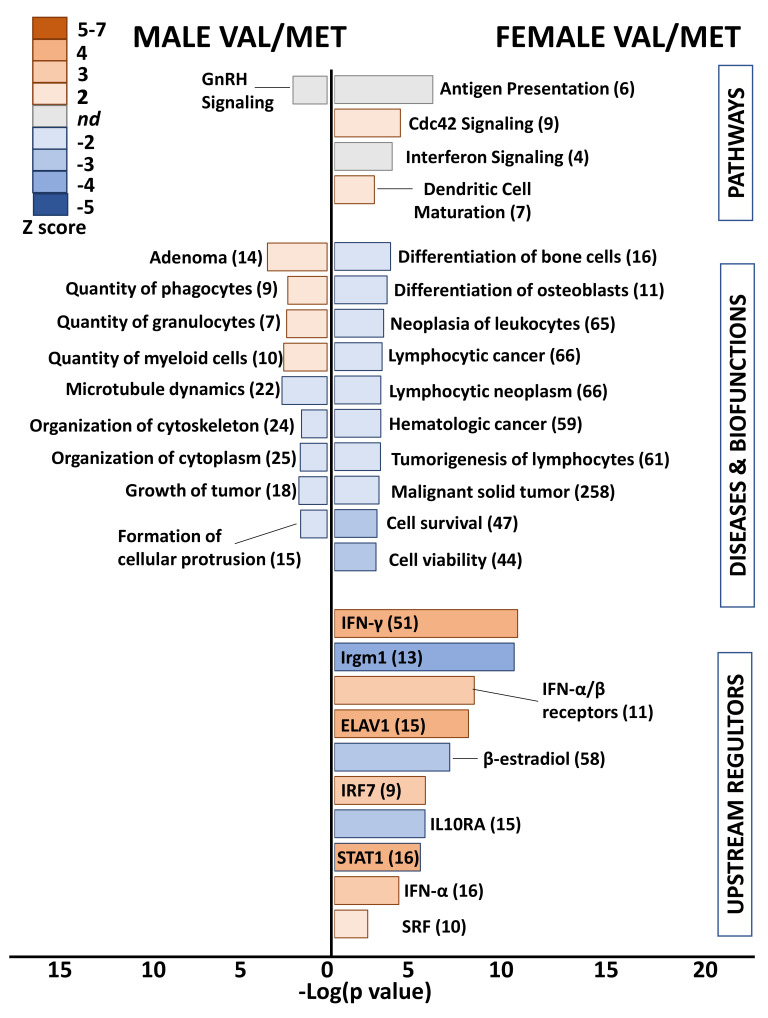
Whole hearts from Val/Met mice exhibit blunted functional profiles loosely related to immune cell proliferation. Bar chart shows enriched pathways (**top** bars), diseases and biofunctions (**middle** bars), and predicted upstream regulators (**bottom**) for whole hearts of male (**left** side) and female (**right** side) Val/Met mice compared to normal Val/Val controls. Bar size reflects significance (shown on *x* axis), and color represents Z score (significance of prediction of directionality), as indicated in the legend. Numbers in parentheses indicate numbers of genes in the listed category that were altered in hearts of either male or female Val/Met mice compared to respective controls.

Differential gene lists with low stringency (uncorrected *p* value < 0.05, >1.25-fold) were used for functional analyses to capture functional patterns that might otherwise be missed due to subtle changes in groups of related genes or low magnitude alterations of genes encoding critical regulatory factors. Even with these reduced filters, an obvious functional pattern was lacking in differential genes of heterozygous Val66Met males (Figure 4). Exceptions were enriched quantity of myeloid cells (10 genes, *p* = 9.2 × 10^−3^, Z score = 2.4), decreased cytoskeleton organization (24 genes, *p* = 3.3 × 10^−4^, Z score = −2.1), and predicted inhibition of the upstream regulator interleukin-1α (IL-1α) (5 genes, *p* = 1.1 × 10^−2^, Z score = −2.2).

Heterozygous Val/Met females were similarly devoid of a functional pattern using a stricter significance cutoff. However, low stringency functional analysis revealed predicted inhibition of lymphocytic neoplasm (65 genes, *p* = 3.6 × 10^−3^, Z score = −2.2) and activation of the Cdc42 (cell division cycle 42) signaling pathway (9 genes, *p* = 4.2 × 10^−4^, Z score = 2.2) as unique to heterozygous females (Figure 4). More interesting were the enriched canonical pathways and predicted upstream regulators related to various immune system processes, including predicted up-regulation of interferons and down-regulation of IL10RA, as was found for Met/Met males. For example, 51 genes downstream of INF-γ were altered in Val/Met females (uncorrected *p* < 0.05, 1.25-fold), resulting in significant enrichment (*p* = 3.8 × 10^−10^) and predicted activation (Z score = 2.6) of this important signaling network. Additionally, as was found for Met/Met males, IL10RA was predicted to be inhibited (15 genes, *p* = 2.2 × 10^−5^, Z score = −3.2) in Val/Met females. Although the functional profile of hearts from heterozygous females included prediction of upstream regulators also found in male homozygous cardiac tissues, there was a substantial differences in significance of overlap (*p* = 6.2 × 10^−28^ for males, *p* = 2.9 × 10^−10^ for females), significance of predicted directionality (Z = 7.5 for males, Z = 3.6), and magnitude of activation or inhibition (e.g., 48 differentially expressed genes downstream of IFN-α in male Met/Met mice, and only 16 IFN-α-inducible genes in female Val/Met mice).

### 2.5. Validation by qPCR

**Figure 5:**
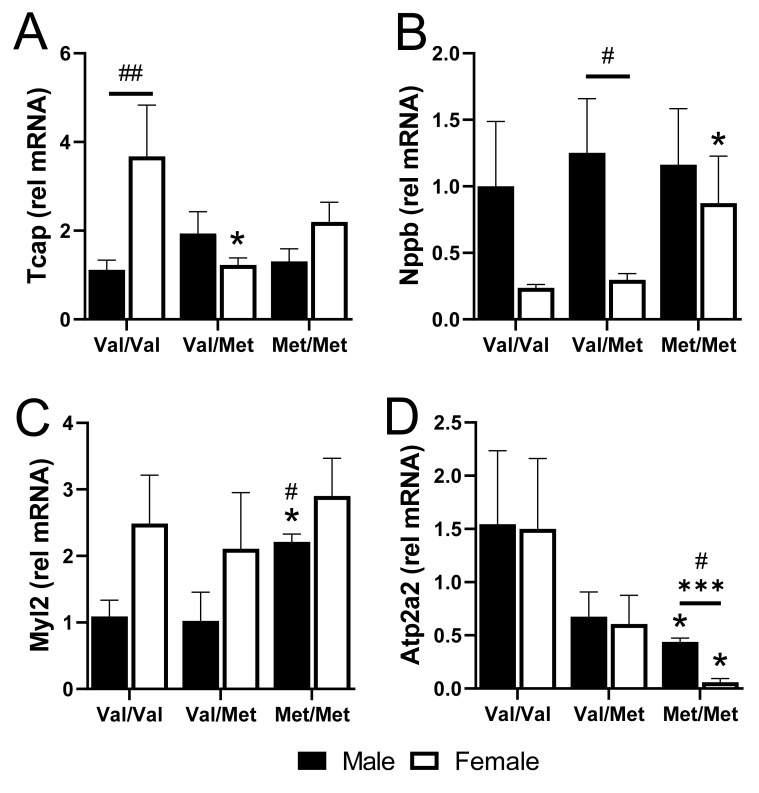
qPCR of select genes validates differences in whole hearts of males and females with and without the Met allele. Bar charts showing mRNA relative to GAPDH of titin-cap, *Tcap* (**A**), natriuretic peptide, *Nppb* (**B**), myosin light chain 2, *Myl2* (**C**), and *Atp2a2* (**D**) for males (black bars) and females (white bars) in each group. *n* = 3 or 4 each group, * *p* < 0.05 for pairwise comparisons between same-sex Val/Met or Met/Met versus respective Val/Val control mice. *** *p* = 0.0003 for pairwise comparison between male and female Met/Met mice. ^#^ *p* < 0.05 and ^##^ *p* = 0.0007 using Mann–Whitney. Relative mRNA was calculated using the ΔΔ CT method with GAPDH as the internal normalization target and male Val/Val as the reference sample.

We validated four cardiac-relevant genes by qPCR, including the gene encoding titin-cap (*Tcap*), the gene encoding natriuretic peptide B (*Nppb*, or BNP), *Myl2* that encodes cardiac myosin light chain 2 (MLC-2v), and ATPase Sarcoplasmic/Endoplasmic Reticulum Ca2+ Transporting 2 (*Atp2a2*), which encodes the cardiac, slow twitch calcium pump, SERCA2. Baseline expression of *Tcap* was higher in Val/Val females than in males but was downregulated specifically in polymorphic female mice with one or both alleles (Figure 5A). Conversely, *Nppb* was 4-fold lower in Val/Val and Val/Met females, compared to males, but was significantly upregulated specifically in female Met/Met mice (Figure 5B). *Myl2*, on the other hand was up-regulated specifically in homozygous males (Figure 5C). Most interesting was the significant down-regulation of *Atp2a2* in both males and females, although most profoundly in Met/Met females (Figure 5D).

## 3. Discussion

For decades, biomedical research primarily used male subjects, largely ignoring the possibility that there could be substantial sex-based differences even in common physiological and pathophysiological processes. In the current era, the importance of sex-based differences in immunologic, cardiovascular, and other diseases has begun to be better appreciated. As such, defining sex-specific parameters are important considerations not only in the clinical setting but also when investigating the role of genetic modifiers in animal models. The results of the current study bring this into focus, as the rs6265 polymorphism leads to disparate expression profiles in male and female mice.

One of the most striking findings in our current study was the lack of gene expression overlap even in the non-polymorphic male and female mice. This has important implications as we move into the era of personalized medicine. As genome data are used to make treatment decisions or tailor therapies to particular individuals, the “expressome” will increasingly need to be considered. Our data demonstrate that the decrease in functional BDNF produced with the rs6265 polymorphism triggers the inflammatory pathway, with a more robust response in homozygous Met/Met male mice. IFN-γ and IL-1β are involved in T helper 1 (Th1) immune responses, while IL-10 is associated with immunoregulation of the Th1 response; thus, in the current study Th1 response is initiated, signifying macrophage activation and inflammation. This is reflected through enhanced expression of various genes involved in the inflammatory process. This represents fertile ground for future investigations into understanding how these mechanisms modulate other disease processes. As an example, we have recently demonstrated that there is decreased cardiomyocyte contractility in male polymorphic mice with a muscular dystrophy cardiomyopathy phenotype [38].

Although female mice did not exhibit a strong inflammatory gene expression profile, key cardiac transcripts were altered, which could contribute to the observed differences in differential cardiac function between female Met carriers, compared to littermate Val/Val controls. Expression of the *Atp2a2* gene, for example, was reduced in male Met/Met mice but essentially obliterated in female Met/Met littermates. *Atp2a2* encodes SERCA2, which is responsible for the lion’s share of Ca2+ reuptake into the SR following cardiac contraction [42] and is thus a major player in diastolic heart function [43]. Reduced SERCA2 might contribute to previously reported differences in the contractile capacity of cardiomyocytes isolated from dystrophic mice with and without the Met allele [38]. However, this supposition requires additional cell-specific studies to confirm this hypothesis. In a human study, low levels of BDNF were associated with cardiovascular diseases and cardiac remodeling [44]. In neuropathological conditions, reduced expression of BDNF is associated with enhanced inflammation and loss of neuroplasticity [45]. Inflammation is characterized with augmented levels of proinflammatory cytokines that trigger activation of leukocytes and have been shown to promote hypocontractility of gut smooth muscle cells [46]. Thus, their increased expression might also affect cardiac or vascular function. Although our results did not show enhanced levels of proinflammatory response in females, there was downregulation of anti-inflammatory genes such as IL-10RA (interleukin 10 receptor subunit alpha) and SOCS1 (suppressor of cytokine signaling), suggesting perturbations in immunoregulatory functions. Future studies will be aimed at identifying the mechanisms responsible for observed transcriptomic and functional differences resulting from presence of the Met allele.

In summary, this is the first report of sex-dependent differences in gene expression profiles of male and female mice with and without the BDNF rs6265 polymorphism. Results suggest that the Met allele predisposes for the development of diastolic dysfunction, perhaps in the context of comorbidities that exacerbate dysfunctional myocardial relaxation. If this presumption is correct, BDNF genetic differences might help to explain opposing results of clinical trials aimed at repairing SERCA2 deficiency using targeted gene therapy [47,48,49,50]. On the other hand, the inflammatory profile observed in male Met/Met mice might confer protection from cardiac remodeling and histopathology associated with BDNF rs6265 polymorphism. Studies such as this one, which include genetic profiling in both males and females, provide insight important for dissecting the underlying pathological mechanisms in the quest for more personalized therapies in the modern era of increasingly individualized medicine.

## 4. Materials and Methods

### 4.1. Animals

This study was carried out in accordance with the National Institutes of Health’s Public Health Service Policy of Humane Care and Use of Laboratory Animals and the Animal Welfare Act. All protocols were approved by the Vanderbilt University Institutional Animal Care and Use Committee under protocol ID# M1700006-00 (27 June 2017). Experiments were performed using appropriate anesthetics, and every effort was made to minimize animal pain and distress. The minimum amount of isoflurane required for animal sedation was used. Transgenic hBDNF^Met^ knock-in allele (Val66Met) mice [51] were produced using heterozygous breeders, and litter mates (8–12 weeks old) were used wherever possible. For all experiments, animals were euthanized with either isoflurane followed by secondary cervical dislocation and subsequent whole heart tissue isolation or via carbon dioxide asphyxiation.

### 4.2. Echocardiography

Transthoracic M-mode echocardiography was performed with a 12-mHz probe (VisualSonics) on conscious mice and on mice anesthetized by inhalation of isoflurane (1–1.5%). LV end-systolic interior dimension (LVID;s), LV end diastolic interior dimension (LVID;d), ejection fraction (EF), and fractional shortening (FS) values were obtained by analyzing data using the Vevo 2100 program. All functional and phenotypic data were obtained from mice that were 8–12 weeks old.

### 4.3. RNA Sequencing

Total RNA was extracted from whole hearts of 8-week-old mice using RNeasy Mini kit (74104, Qiagen, Germantown, MD, USA) as per the manufacturer’s instructions. RNA integrity was confirmed using Agilent Bioanalyzer. RNA sequencing (RNA-Seq) was performed by the Vanderbilt Technologies for Advanced Genomics (VANTAGE) core. RNA libraries were constructed using the Illumina TruSeq Stranded Total RNA kit (Illumina Inc., San Diego, CA, USA). Libraries were sequenced using Illumina HiSeq 3000 on paired-end-150 flow cell runs at ~32 M PF reads per sample. Raw reads (fastq files) were uploaded to the Partek Flow server Build version 10.0.21.0621 (Partek Incorporated, St. Louis, MO, USA) and pre-alignment quality assessment was performed. Mean base-call quality scores were above Phred-like values of 36 in all positions of all samples, and no hard trimming of the reads was necessary. Sequences were aligned to the mm10 assembly of the mouse genome using STAR 2.5.3a and resulting summary of reads filtered (<80) and quantified at the gene level to Ensemble transcripts 83 using Partek’s expectation maximization (E/M) annotation model. Gene counts were normalized to total read count per sample and then log-transformed (with an offset of 0.0001). To identify differentially expressed genes, statistical analysis was performed using Partek’s Gene Specific Analysis (GSA) multimodal estimation algorithm, which identifies the statistical model that is the best for each gene among all the selected models (lognormal, Poisson, etc.), and then uses that best model to calculate *p*-value and fold change. Statistical analyses (including correction for multiple hypothesis testing) for identification of overrepresented ontologies, functions, and pathways were performed using Ingenuity Pathway Analysis software (Qiagen).

### 4.4. Validation by qPCR

Total RNA from whole hearts was converted to cDNA with iScript (Bio-Rad). Pre-validated primers for murine *Myl2*, *Tcap*, *Nppb*, and *Atp2a2* were purchased from Qiagen. Relative gene expression for each receptor was assessed using iTaq Universal SYBR Green Supermix (Bio-Rad) in a Bio-Rad CFX instrument, according to the manufacturer’s protocol. Briefly, ~200 ng of cDNA was mixed with 2X Supermix mix, RNase-free water, and 1 µM of primers, for a total reaction volume of 10 µL. A typical protocol was included for polymerase activation at 95 °C for 30 s, and 40 cycles as follows: denaturation (5 s at 95 °C), annealing/extension (30 s at 60 °C), followed by melt-curve analysis. The comparative threshold method was used to calculate fold-differences in Excel. Pre-validated mouse GAPDH primers served as internal controls to normalize target gene expression across different samples. At least 4 biological and 2 technical replicates were included to ensure reproducibility.

### 4.5. Statistics

All data are expressed as means ± SEM. Statistical comparisons made between 2 variables were performed using two-way ANOVA or the Student’s t test (for pairwise comparisons) or Mann-Whitney for multiple comparisons. Comparisons between more than 2 variables were performed using one-way ANOVA with a Tukey’s post hoc test. *p* values of less than 0.05 were considered statistically significant. For RNASeq, Partek was used to perform pairwise comparisons of average group values, and only transcripts that resulted in a fold-change of at least 1.5 and *p* value of less than 0.05 were considered significantly altered.

## 5. Limitations

This study was limited by issues inherent to the hBDNF transgenic mouse model, in which the mutated human BDNF was expressed and subsequently compared to control mice in which normal mouse BDNF was expressed. Mice were bred in heterozygous pairs, and littermates used throughout to minimize potential bias. Another limitation was the lack of specificity regarding speculative interpretations of observed variations, because whole hearts were used rather than isolations of specific cardiac cell populations.

## 6. Translational Perspective

The rs6265 polymorphism modifies baseline cardiac function in female mice and differentially alters baseline cardiac transcription in males versus females; thus, it may represent a novel risk factor for worse outcomes in cardiovascular disease or confer a benefit for some etiologies of cardiology. Sex-based differences represent a potential confounder for studies of rs6265 as a biomarker or modifier of disease risk.

## Figures and Tables

**Table 1:** Echocardiograph Assessment of Male and Female Mice with and without the rs6265 Allele.

	Val/Val	*p*	Val/Met	*p*	Met/Met	*p*
	Male (*n* = 9) Female (*n* = 6)		Male (*n* = 17) Female (*n* = 13)		Male (*n* = 6) Female (*n* = 6)	
HR (BPM)	681 ± 41.0	0.578	653 ± 39.1	0.232	653 ± 39.5	0.227
	689 ± 15.1		675 ± 25.5		654 ± 29.4	
LViD;s (mm)	1.81 ± 0.32	0.006	1.53 ± 0.29	0.752	1.85 ± 0.43	0.105
	1.30 ± 0.28		1.57 ± 0.23		1.45 ± 0.25	
LViD;d (mm)	3.20 ± 0.32	0.047	2.99 ± 0.26	0.460	3.29 ± 0.34	0.210
	2.75 ± 0.40		3.08 ± 0.29		3.02 ± 0.27	
ESV (μL)	10.5 ± 4.7	0.006	7.2 ± 4.3	0.739	11.4 ± 5.2	0.110
	4.54 ± 2.3		7.7 ± 1.9		6.1 ± 3.14	
EDV (μL)	41.5 ± 10.1	0.037	35.2 ± 8.6	0.388	44.6 ± 10.4	0.201
	29.3 ± 9.8		38.4 ± 7.54		36.2 ± 7.8	
SV (μL)	31.1 ± 6.0	0.122	28.1 ± 5.0	0.305	33.2 ± 5.8	0.396
	24.7 ± 7.8		30.7 ± 6.0		30.1 ± 5.3	
LVEF (%)	75.67 ± 6.0	0.002	80.8 ± 5.58	0.997	75.8 ± 8.4	0.078
	85.24 ± 3.4		80.8 ± 3.4		84.1 ± 4.7	
LVFS (%)	43.7 ± 5.4	0.002	49.3 ± 5.2	0.993	44.14 ± 9.1	0.089
	53.3 ± 4.4		49.3 ± 4.6		52.7 ± 4.7	

## Data Availability

Raw and processed sequencing data were deposited in the NCBI Gene Omnibus database under the unique identifier Accession GSE178685.
